# Barriers and enablers to centre-based pulmonary rehabilitation for patients with chronic obstructive pulmonary disease in low- and middle-income countries: a systematic review

**DOI:** 10.7189/jogh.15.04255

**Published:** 2025-09-19

**Authors:** Yue Lei Lim, Julia Patrick Engkasan, Jayakayatri Jeevajothi Nathan, Hilary Pinnock, Ee Ming Khoo, Monsur Habib, Soo Chin Chan, RESPIRE Collaboration

**Affiliations:** 1 Department of Rehabilitation Medicine, Faculty of Medicine, Universiti Malaya, Kuala Lumpur, Malaysia; 2 Department of Primary Care Medicine, Faculty of Medicine, Universiti Malaya, Kuala Lumpur, Malaysia; 3 NIHR Global Health Research Unit on Respiratory Health (RESPIRE), Usher Institute, University of Edinburgh, Edinburgh, UK; 4 Bangladesh Primary Care Respiratory Society, Khulna, Bangladesh; 5 College of Medicine and Veterinary Medicine, Usher Institute, University of Edinburgh, Edinburgh, UK

## Abstract

**Background:**

Chronic obstructive pulmonary disease (COPD) is a leading cause of mortality and morbidity in low- and middle-income countries (LMICs). Despite the proven benefits of pulmonary rehabilitation (PR) for patients with COPD, its referral, uptake, and completion rates remain low. This systematic review aimed to identify the barriers and enablers to centre-based PR among patients with COPD in LMICs.

**Methods:**

We searched PubMed, Web of Science, Cumulative Index to Nursing and Allied Health Literature, and Scopus databases from their inception to September 2023 and updated in May 2025. Studies involving patients with COPD, their caregivers, and healthcare professionals (HCPs), were included if they reported barriers or enablers to centre-based PR in LMICs. Data were extracted based on the socio-ecological model of health behaviour, and a narrative synthesis was conducted.

**Results:**

Five articles met the inclusion criteria, comprising four quantitative and one qualitative study, involving 1544 patients with COPD, 11 caregivers, and 84 HCPs, which were conducted in Iran, China, Colombia, and Brazil. The most frequently identified barrier to PR was personal financial constraints. Other frequently reported barriers included symptom severity of COPD, lack of family and social support, inadequate competency of HCPs, and logistical challenges. Enablers to PR included higher proficiency of HCP, higher personal and family income, higher educational levels, better patient awareness of PR, and awareness programmes.

**Conclusions:**

Barriers and enablers to PR referral, uptake, and completion in LMICs were identified at multiple levels: intrapersonal, interpersonal, organisational, community, and policy. Some factors were common to both LMICs and high-income countries, such as frequency of hospitalisation, social support, HCP knowledge and skills, logistical challenges, and awareness programmes, but personal financial constraints were a unique barrier to LMICs. To improve existing PR services or to effectively implement new PR programmes, these factors need to be considered.

**Registration:**

PROSPERO: CRD42024528467.

Chronic obstructive pulmonary disease (COPD) is a prevalent and growing global health issue, driven primarily by factors such as smoking and exposure to biomass fumes [1,2]. It significantly impacts morbidity and mortality rates, and despite advancements in management, no curative treatment exists. Approximately 400 million people worldwide are affected by COPD, with 80% residing in low- and middle-income countries (LMICs) [3]. The prevalence of COPD in LMICs is expected to rise compared to high-income countries (HICs), increasing the economic burden [1,4].

Patients with COPD often report dyspnoea as their most distressing symptom [5,6]. The disease is also associated with muscle weakness and exercise intolerance [7], creating a vicious cycle of physical inactivity, deconditioning, and dyspnoea-related fear [8–10]. These significantly limit social participation and diminish quality of life.

Pulmonary rehabilitation (PR) is a well-established, non-pharmacological intervention for COPD. The American Thoracic Society and European Respiratory Society define PR as *a comprehensive intervention based on a thorough patient assessment followed by patient-tailored therapies, which include, but are not limited to, exercise training, education, and behaviour change, designed to improve the physical and psychological condition of people with chronic respiratory disease and to promote long-term adherence to health-enhancing behaviours* [11]. It alleviates symptoms such as dyspnoea and fatigue, improves exercise tolerance [12,13], and enhances psychological well-being and quality of life [14–16]. Additionally, it reduces exacerbations, hospitalization, and re-hospitalisation rates, and mortality [17–20]. The reduction in exacerbations can slow clinical deterioration and yield significant cost savings.

Despite the proven effectiveness of PR, referral rates (4–33%) [21,22], uptake rates (7–68%) [23,24], and completion rates (18–57%) [23,25] remain low. Previous studies have identified barriers and enablers to PR referral, uptake, and completion, but most were conducted in HICs. To our knowledge, no systematic review has been published on this topic in LMICs. Therefore, this review aimed to identify the barriers and enablers to PR referral, uptake, and completion among patients with COPD in LMICs.

## METHODS

### Literature search

The search strategy was organised around four key concepts: COPD, PR, referral/uptake/completion, and LMICs (Table S1 in the **Online Supplementary Document**). Search terms were adapted to the specific syntax of each database to maximise the number of relevant studies. Four electronic databases (PubMed, Web of Science, Cumulative Index to Nursing and Allied Health Literature (CINAHL), and Scopus) were searched for articles from inception until September 2023 (Table S2 in the **Online Supplementary Document**). The search was updated pre-publication in May 2025. All the search results were imported into EndNote for article management. In addition to database searches, Google was used to identify further references not indexed in traditional academic databases. A manual search of reference lists from included studies was also conducted to identify additional relevant studies.

This systematic review followed the reporting format in accordance with the PRISMA flow diagram [26].

### Eligibility criteria

The inclusion criteria for this study were:

1. studies involving patients with COPD, as defined by the investigators, healthcare professionals (HCPs) managing patients with COPD and/or PR, and family members/carers of patients with COPD

2. studies conducted in LMICs

3. studies of centre-based PR programmes

4. studies reporting barriers or enablers to referral, uptake, or completion of PR

5. qualitative or quantitative studies

6. only studies published in English were included.

The exclusion criteria were:

1. studies involving individuals with diagnoses other than COPD

2. studies conducted in HICs

3. studies of home-based PR programmes

4. studies of telerehabilitation

5. studies that did not report on referral, uptake, or completion of PR

6. conference abstracts, editorials, and reviews.

Duplicates were identified and removed before screening the titles and abstracts. Two researchers (SCC and YLL) independently screened the titles and abstracts based on the inclusion and exclusion criteria to identify relevant studies. Articles were classified as include, maybe, or exclude. The full texts of all articles classified as ‘maybe’ or ‘include’ were retrieved. SCC and YLL independently assessed the full texts for eligibility and articles for inclusion were identified. Consensus was reached through discussion between SCC and YLL. In cases of disagreement, a third researcher (JPE) was asked and a final decision made.

The quality of the included studies was assessed using the quality assessment scale from Keating et al. [27], which was adapted to assess the relevant aspects of both qualitative and quantitative research, which included quality of reporting, external validity, bias, and confounding factors (Table S3 in the **Online Supplementary Document**). It was used to assess both qualitative and quantitative studies on barriers to the uptake and attendance of PR in patients with COPD [27,28]. The quality of each included article was independently evaluated by SCC and YLL. Each item in the scale was scored as 1 for ‘Yes’, 0.5 for ‘Unclear’, and 0 for ‘No’. Any discrepancies were resolved through discussion to reach a consensus.

### Data extraction and data analysis

Data extraction was conducted independently by SCC and YLL using a standardised extraction table developed to capture the study characteristics, including the year and location of the study, study population, and study design. Data on barriers and enablers to the referral, uptake, and completion of PR were then extracted and mapped according to the socio-ecological model of health behaviour [29,30]. This model considers intrapersonal, interpersonal, organisational, community, and policy factors as influences on an individual’s health behaviour.

## RESULTS

### Literature search

**Figure 1:**
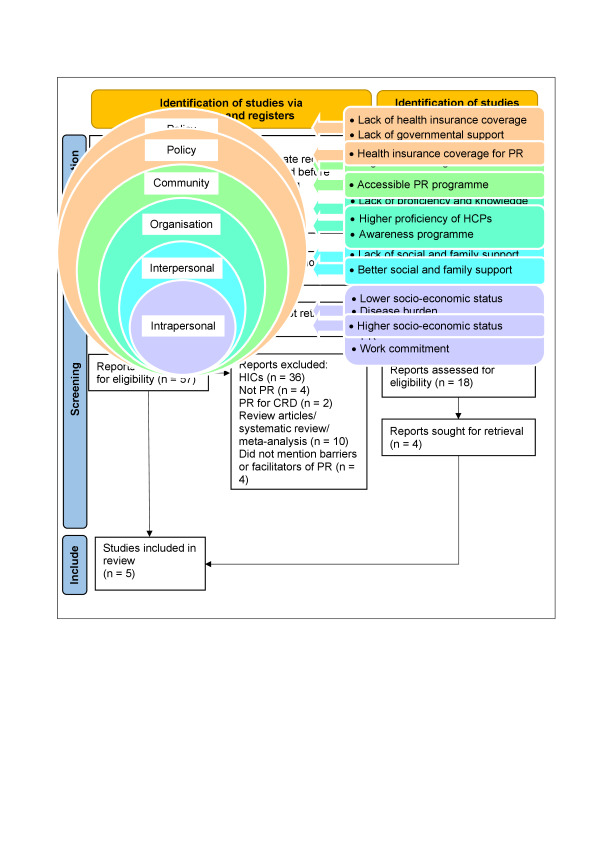
PRISMA flow diagram.

A total of 4291 records were retrieved, 4287 from electronic database searches and four from Google web searches. After removing 407 duplicates, 3884 records were screened for title and abstract. Of these, 3816 were excluded, leaving 68 records for full-text assessment. Among these, seven were abstracts only and 61 full texts were reviewed. Four articles met the inclusion criteria. An updated search in May 2025 identified 729 additional articles. Following a similar selection process, one additional article was included, bringing the total number of articles included in this systematic review to five (**Figure 1**).

### Characteristics of the included studies

The five included studies involved 1544 patients with COPD, 11 caregivers, and 84 HCPs and were conducted in Brazil, China (two articles), Iran, and Colombia [31–35] between 2019–2021. Four studies were cross-sectional and one was a qualitative study [32] and the remaining four being questionnaire-based. [31,33–35]. Three studies [33–35] included only patients with COPD, one study [31] included only HCPs, and another study [32] included patients and caregivers of patients with COPD, and HCPs (**Table 1**). One study reported on referral of PR [31], three studies reported on uptake of PR [32,34,35], and two studies reported on attendance and completion of PR in patients with COPD [32,33] (Table S4 in the **Online Supplementary Document**).

**Table 1:** Characteristics of the included studies

Study, Quality assessment score	Duration of study	Country	Study design	Population of patients and caregivers	Population of HCPs
Gushken et al. [31], 7	May–October 2019	Brazil	Cross-sectional study, questionnaire-based	-	72 physicians (internal medicine, geriatrics, cardiology, pulmonology, thoracic surgery)
Xie et al. [34], 9.5	16 April 2019–21 January 2020	China	Cross-sectional study, questionnaire-based	1138 patients with COPD aged >40 y, COPD diagnosed by GOLD guidelines	-
Yao et al. [35], 12	1 July 2020–30 October 2020	China	Cross-sectional study, questionnaire-based	237 patients with COPD aged >40 y, COPD diagnosed by GOLD guidelines	-
Sami et al. [32], 9.5	January 2019–October 2020	Iran	Qualitative descriptive study, semi-structured personal interviews	19 patients with COPD, 11 caregivers of patients with COPD	12 HCPs
Betancourt-Peña et al. [33], 11	August 2020–April 2021	Colombia	Cross-sectional study, questionnaire-based	150 patients with COPD	-

COPD – chronic obstructive pulmonary disease, GOLD – Global Initiative for Obstructive Lung Disease, HCPs – healthcare professionals

### Quality assessment

The quality assessment scores ranged from 7–12. Most studies described the data collection methods, employed systematic data collection, and used appropriate data analysis methods. Some studies did not clearly define the researcher-patient relationship [31,32,34] nor establish validity [31,33,34] (**Table 1**; Table S3 in the **Online Supplementary Document**).

### Barriers to referral, uptake, and completion of PR

**Figure 2:**
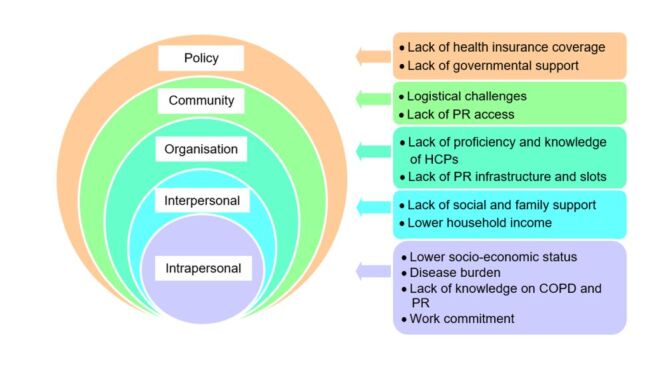
Barriers to referral, uptake, and completion of PR. PR – pulmonary rehabilitation.

Barriers were identified at multiple levels influencing referral, uptake, and completion of PR (**Figure 2**, **Table 2**). Gushken et al. [31] reported 25% of physicians never referred patients to PR, while another 18% of physicians rarely referred. Among physicians who treated patients with COPD, the three most common barriers to PR referral were financial concerns (79%), distance to the PR centre (63%), and lack of social support (43%). Other barriers included incompatible work schedules, lack of available slots in PR programmes, patient refusal, and lack of knowledge of PR.

**Table 2:** Barriers to referral, uptake, and completion of PR

Variable	Barriers to referral	Barriers to uptake	Barriers to completion	Number of articles/number of included articles
**Intrapersonal level**				
Socio-economic status	Unable to afford PR [31]	Lower monthly income [34]; heavy financial burden of medications and PR services [32,35]	Could not afford healthcare payments [33]	5/5
Symptoms and control of COPD	Breathlessness [31]	Exacerbation of COPD [32]; Exacerbation of COPD which led to hospitalisation [32]; Fewer hospitalisation per year [34]		3/5
Severity and chronicity of COPD		COPD requires long-term treatment and care [32]; Lower GOLD classification [34]		2/5
Belief and motivation	Refusal for PR [31]	Frustration [32]		2/5
Work commitment	Incompatible work schedule with PR programme [31]			1/5
Awareness of PR		Low awareness of PR [35]		1/5
Knowledge on COPD and PR		Lack of knowledge on COPD and PR [32]		1/5
General condition	Frailty [31]	Fatigue [32]		2/5
Psychosocial aspect		Inability to cope with COPD [32]; Inability to regain independence [32]; Psychological stress [32]		1/5
Alcohol consumption		Frequent alcohol consumption [35]		1/5
**Interpersonal level**				
Family/ social support	Lack of social support from friends and family [31]	Family members imposed psychological stress [32]; Lack of social support from friends and family [32,35]		3/5
Household socio-economic status		Lower household income [34]		1/5
Family’s knowledge on COPD and PR		Lack of knowledge of family on COPD and PR [32]		1/5
**Organisational level**				
Efficiency of healthcare systems			PR was not available during evenings and nights [32]; Inefficient PR record system [32]; Limitations of services in the clinic [32]; Lack of continuity of care outside the clinical setting [32]	1/5
Institutional or infrastructure problem(s)	Lack of available PR slots [31]		Lack of PR equipment [32]	2/5
Practice and competency of HCPs	Lack of knowledge on the benefits of PR [31]; Prescribed only home exercises [31]; Did not know the location of PR centres [31]	Worse PR skills [35]	Lack of clear job scope for PR team members [32]; Lack of knowledge on PR [32]; Incompetent PR team members [32]; Lack of coordination among PR team members [32]	3/5
Attitude of HCPs			Lack of patient-centredness and holistic approach [32]; Lack of motivation for patient care [32]	1/5
**Community level**				
Access to PR		Lack of PR centres [32]		1/5
Distance from home to PR centres	Long distance from home to PR centres [31]	Long distance from home to PR centres [32]		2/5
Convenience of transportation		Inconvenient transportation [35]		1/5
Public education programme		Lack of public education programme [35]		1/5
**Policy level**				
Health insurance coverage for PR	Lack of health insurance coverage for PR [31]		Lack of health insurance coverage for PR [32]	2/5
Governmental support		Lack of support from the government [32]		1/5
Clinical guidelines			Lack of PR protocols [32]	1/5

COPD – chronic obstructive pulmonary disease, GOLD – Global Initiative for Obstructive Lung Disease, HCPs – healthcare professionals, PR – pulmonary rehabilitation

The overall uptake rate of PR from Xie et al. [34] was approximately 25%. Nearly half of the participants felt they needed PR, while the remainder either did not feel the need or were unsure. Lack of understanding about PR was common among patients with COPD. In a questionnaire survey with 18 questions related to PR, the mean number of correct answers was only 7.8 [34]. Additionally, more than 80% of patients believed that PR should be stopped as soon as breathlessness occurred, and fewer than one-third understood that starting PR immediately after discharge from an acute COPD exacerbation would be effective [34].

Betancourt et al. [33] showed adherence to PR was good (completed at least 85% of the sessions) in more than 50% of patients and poor (completed less than 35% of the sessions) in 18%. This study also identified the most common reasons for discontinuing PR: clinical and safety issues (17.3%), economic factors (10.7%), and changes in location due to insurance (6.7%).

### Enablers to referral, uptake, and completion of PR

**Figure 3:**
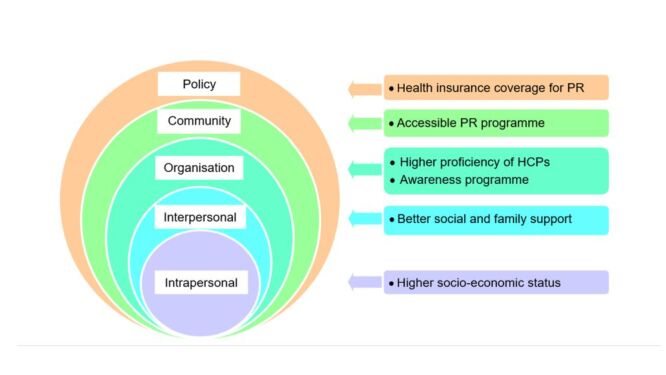
Enablers to referral, uptake, and completion of PR. PR – pulmonary rehabilitation.

The enablers to the referral, uptake, and completion of PR in patients with COPD were identified (**Figure 3**, **Table 3**). Three out of the five studies identified HCP competency as a key enabler of referral, uptake, and completion of PR. Two out of the five studies cited better socio-economic status, accessible healthcare systems, and health insurance coverage as facilitators.

**Table 3:** Enablers to referral, uptake, and completion of PR

Variable	Enablers to referral	Enablers to uptake	Enablers to completion	Number of articles/ Number of included articles
**Intrapersonal level**				
Gender			Male [33]	1/5
Socio-economic status		Higher monthly income [34,35]; Higher educational level [35]		2/5
Control of COPD		More hospitalisations per year [34]		1/5
Severity and chronicity of COPD		Higher GOLD classification [34]		1/5
Awareness of PR		Better awareness of PR [35]		1/5
Alcohol consumption		Does not consume alcohol [35]		1/5
**Interpersonal level**				
Family/ social support		Better support from friends and family [35]		1/5
Household socio-economic status		Higher household income [34]		1/5
**Organisational level**				
Healthcare systems	Telemedicine [31]; Partnership with other PR centres [31]		Expand PR services to home settings [32]	2/5
Competency of HCPs	Awareness campaigns and online or printed educational materials [31]	Better PR skills [35]	Strong collaboration among PR team members [32]	3/5
**Community level**				
Convenience of transportation		Convenient transportation [35]		1/5
Public education programme		More public education programme [35]		1/5
**Policy level**				
Health insurance coverage for PR	Increase coverage for PR [31]		Subsidised healthcare [33]	2/5

COPD – chronic obstructive pulmonary disease, GOLD – Global Initiative for Obstructive Lung Disease, HCPs – healthcare professionals, PR – pulmonary rehabilitation

The multivariate analysis of patients with COPD by Xie et al. [34] found that higher Global Initiative for Obstructive Lung Disease (GOLD) classification, more hospitalisations, and higher personal and family income were enablers for PR uptake. Among patients who recognised the need for PR, higher personal income, family income, and more hospitalisations per year were linked to higher attendance rates. Conversely, for patients who did not believe they needed PR or were uncertain about its necessity, only more hospitalisations per year served as a protective factor for PR participation [34].

Yao et al. [35] reported that factors such as personal awareness of PR therapy, awareness programmes, convenient transportation, abstinence from alcohol, proficiency of HCPs, and family support were significantly associated with patients' intention to accept PR services. Gushken et al. [31] found that increasing awareness and education and delivering PR via telemedicine (74 and 46% of physicians’ opinions respectively) could enhance PR referrals.

## DISCUSSION

The most frequently reported barriers to PR were personal financial constraints. Additional barriers to PR included symptom severity of COPD, lack of family and social support, inadequate competence of the HCPs, and logistical challenges. Conversely, several enablers to PR were identified. These included higher proficiency of HCPs, higher personal and family income, higher educational levels, and awareness programmes.

### Barriers to PR

Our systematic review identified financial constraints as the most common barrier to PR therapy. These findings align with previous studies indicating that lower monthly and household income levels are associated with reduced health-seeking behaviour [36]. Patients with COPD face substantial direct costs, such as treatment, medication, consultation fees, and hospitalisation, alongside indirect costs, including loss of employment, mandatory retirement, and sick days. For instance, the direct healthcare cost may be up to one third of the average income *per capita* in China [37], which imposed significant financial burden on patients with COPD. Additional financial burden of PR such as transportation cost, service fees, and time off work can be overwhelming for those struggling to make ends meet.

Two previous systematic reviews in HICs did not identify personal income or affordability as significant barriers to PR [28,38]. This highlights a stark discrepancy between the challenges faced by LMICs and HICs. A cost-effectiveness study conducted in the USA showed that PR resulted in net savings of approximately 6000 USD per patient, in addition to improving quality-adjusted life expectancy [39]. Demonstrating similar cost-saving outcomes in LMICs could help secure essential financial support for patients through government programmes or insurance providers.

We also found that logistical issues and lack of social support are common barriers to PR, consistent with findings from HICs [28]. Patients with COPD often struggle with daily activities at home, and this difficulty is exacerbated when traveling outside their homes [10]. Consequently, family support becomes essential not only for activities of daily living but also for transportation to PR sessions [31]. Factors such as distance to the PR centres, travel time, and costs are significant considerations, particularly for patients from lower socioeconomic backgrounds [40,41].

Both frequent and few hospitalisations due to COPD were associated with reduced uptake of PR. This dual trend suggests distinct subgroups of patients face different barriers to PR uptake. It was also reflected in other studies that reported how perceptions of severity affected PR uptake [24,42]. Patients who are too well may not perceive PR as being required or not being beneficial [43]. Those with more severe disease may be hindered by practical barriers of mobility and relying on others to support their travel [10]. Additionally, some HCPs did not refer patients to PR, citing the belief that the patient’s condition was either not severe or too advanced for PR to be effective [38].

Xie et al. [34] highlighted that many patients were apprehensive about the safety and effectiveness of starting PR shortly after discharge, despite evidence showing that early initiation is safe, effective, and reduces re-hospitalisations and mortality [19]. Furthermore, patients with inadequate knowledge of the benefits of PR may be less willing to participate in PR programmes [34,35].

Similar to findings in HICs, our review identified HCPs’ lack of proficiency and knowledge of delivering PR as a significant barrier. Many HCPs reported limited education about PR, had insufficient equipment, and absence of clear PR guidelines [32,41]. Previous studies had shown that COPD guidelines are less available in LMICs, with an estimated 1.93 billion people living in countries without a national COPD guideline [44,45]. Hence, HCPs who lack confidence in managing PR or remained unconvinced of its benefits may struggle to encourage patients’ participation in PR programmes [46–48].

### Enablers to PR

In line with our earlier finding that cost was a significant barrier to PR, higher personal and family income was identified as a key enabler for the uptake of PR. Patients and family members with higher income levels are better able to afford the additional costs associated with attending regular PR programmes. Furthermore, patients with COPD with higher income and educational levels tend to have greater health literacy, enabling them to advocate more effectively for their own health [49].

Self-management interventions, with modifications to adapt to socio-economically deprived populations, have been proven to improve health outcomes [50]. These should be emphasised and incorporated into part of the management of patients with COPD in LMICs, with modifications such as individualised sessions and simplified instructions to address differences in health literacy, educational background, and cultural differences [51,52].

The competency of HCPs delivering PR was highlighted as an important factor in increasing referral, uptake and completion rates. Those HCPs with better knowledge, motivation, and teamwork skills are more likely to provide holistic treatment, which enhances patient satisfaction and adherence to treatment plans. Additionally, they are able to offer tailored advice specific to individual patients rather than relying on generic information about PR. Implementing a COPD discharge care bundle for all patients, which includes PR referral, may improve COPD knowledge, increase PR uptake, and reduce rehospitalisation rates [53,54]. For remediation of barrier of lack of equipment for PR, supervised PR with minimal equipment had been shown to improve exercise endurance and quality of life [55]. In addition, telerehabilitation and home-based PR programmes are other feasible options for improving PR [56,57]. They had been shown to improve exercise capacity and quality of life without requiring patients to commute to PR centres [58,59].

Awareness programmes were also frequently reported as enablers of PR referral and uptake. A previous systematic review of 28 observational studies found that the median referral rate was only 16% [38], underscoring a significant lack of recognition of potential benefits of PR. Educational programmes for HCPs, combined with awareness campaigns, lectures, educational booklets and videos, and simulations aligned with COPD guidelines, are essential for improving their knowledge of COPD management and PR [46,60,61]. These initiatives can ultimately improve patient care. At the community level, COPD awareness programmes are also valuable for increasing patients’ awareness of PR and fostering family and social support [35].

### Strengths and limitations

Using the socio-ecological model of health behaviour, we could depict the complex interplay of elements influencing PR and provide a framework to help researchers and policymakers in implementing PR more effectively. This review also highlighted the unique challenges faced by LMICs compared to HICs, underscoring the need for high-quality local data to address region-specific issues. This is the first review to systematically analyse the barriers and enablers to PR for patients with COPD exclusively in LMICs.

However, our findings are based on only five eligible studies from three regions (the Middle East, East Asia, and Latin America), all of which were conducted in upper or lower middle-income countries. None were from low-income countries (LICs) so our insights into barriers and enablers in the middle-income country context may not be applicable in LICs where the financial, health service, social support, and transport infrastructure barriers may be even more challenging. The lack of published articles on PR in LICs may represent a lack of health system provision or limited research capacity in applied research. A global lack of priority attached to respiratory non-communicable disease [62] and chronic under-funding of respiratory health service research [63] will exacerbate this situation. Another limitation of this review is that the adopted quality assessment scale was not formally validated, which may influence the robustness of the quality assessment findings.

### Future research

There is a notable gap in PR research in LMICs, particularly in LICs. Future research should prioritise generating evidence from LIC to better inform equitable PR service development globally.

As low socio-economic status was identified as the most common barrier to PR in LMICs, conducting cost-benefit analyses is essential to highlight the potential cost savings associated with patients attending and completing PR. Demonstrating a favourable cost-effectiveness would be valuable for engaging both public and private sectors to enhance the availability and utilisation of PR programmes.

## CONCLUSIONS

We identified personal financial constraints as a unique barrier in LMICs while other barriers and enablers to referral, uptake, and completion of PR in LMICs such as frequency of hospitalisations, logistical challenges, knowledge and skills of HCPs, social support, and awareness programmes were similar to HICs. To improve and establish new PR programmes, these factors need to be considered.

## Additional material


Online Supplementary Document
