# Supplementary material for: Barriers and enablers to centre-based pulmonary rehabilitation for patients with chronic obstructive pulmonary disease in low- and middle-income countries: a systematic review
Source: J Glob Health. 2025 Sep 19;15:04255. doi: 10.7189/jogh.15.04255 (PMC12447018; doi:10.7189/jogh.15.04255)
Supplement: Online Supplementary Document [file jogh-15-04255-s001.pdf]

Supplement to: **Lim YL, Patrick Engkasan J, Jeevajothi Nathan J, Pinnock H, Khoo EM, Habib GMM, Chan SC; RESPIRE Collaboration. Barriers and enablers to centre-based pulmonary rehabilitation for patients with chronic obstructive pulmonary disease in low- and middle-income countries: a systematic review. J Glob Health. 2025;15:04255.**

Table S1: Search terms and strategy

No.	Concepts	Search Terms
**1.**	COPD	"chronic obstructive pulmonary disease" OR "COPD" OR "chronic obstructive airway disease" OR "COAD" OR "chronic obstructive lung disease" OR "COLD" OR "chronic airflow obstruction" OR "obstructive lung disease" OR "obstructive pulmonary disease" OR "obstructive airway disease"
**2.**	PR	"pulmonary rehabilitation" OR "pulmonary exercise" OR "breathing exercise" OR "respiratory therapy" OR "physical therapy"
**3.**	LMICs	"low- and middle-income country" OR "low income country" OR "middle income country" OR "developing country" OR "developing nation"
**4.**	Referral, uptake, complete	"refer" OR "assign" OR "introduce" OR "prescribe" OR "uptake" OR "recruit" OR "participate" OR "attend" OR "complete" OR "finish" OR "adhere"

Table S2: The search terms according to the electronic databases

Database	Search terms
PubMed	("Pulmonary Disease, Chronic Obstructive"[Mesh]) OR ("Lung Diseases, Obstructive"[Mesh])) OR (Chronic obstructive pulmonary disease*)) OR (Chronic obstructive airway disease*)) OR (Chronic obstructive lung disease*)) OR (COPD)) OR (COAD)) OR (COLD)) OR (Chronic airflow obstruction*)) OR (Obstructive lung disease*)) OR (Obstructive pulmonary disease*)) OR (Obstructive airway disease*)<br>AND<br>("Respiratory Therapy"[Mesh]) OR ("Breathing Exercises"[Mesh])) OR ("Respiratory Therapy"[Mesh])) OR ("Physical Therapy Modalities"[Mesh])) OR (Respiratory Therap*)) OR (Pulmonary Rehabilitat*)) OR (Cardiopulmonary Rehabilitat*)) OR (Pulmonary exercis*)) OR (Breathing exercis*)) OR (Physical therap*)<br>AND<br>("Poverty"[Mesh]) OR ("Developing Countries"[Mesh])) OR (Developing Countr*)) OR (Developing Nation*)) OR (LMIC*)) OR (MIC*)) OR (LIC*)) OR (Low and Middle Income)) OR (Low-and-Middle Income)) OR (Low Income)) OR (Low-income)) OR (Middle Income)) OR (Middle-income)) OR (Lower-Middle-Income)) OR (Lower Middle Income)) OR (Low Middle Income)) OR (Low-Middle-Income)<br>AND

	(Refer*) OR (Assign*)) OR (Introduc*)) OR (Prescrib*)) OR ((((Uptak*) OR (Recruit*)) OR (Participat*)) OR (Attend*))) OR (((Complet*) OR (Finish*)) OR (Adhere*))
Web of Sciences	Chronic obstructive pulmonary disease* OR Chronic obstructive airway disease* OR Chronic obstructive lung disease* OR COPD OR COAD OR COLD OR Chronic airflow obstruction* OR Obstructive lung disease* OR Obstructive pulmonary disease* OR Obstructive airway disease*<br>AND<br>Respiratory Therap* OR Pulmonary Rehabilitat* OR Cardiopulmonary Rehabilitat* OR Pulmonary exercis* OR Breathing exercis* OR Physical therap*<br>AND<br>Developing Countr* OR Developing Nation* OR LMIC* OR MIC* OR LIC* OR Low and Middle Income OR Low-and-Middle Income OR Low Income OR Low-income OR Middle Income OR Middle-income OR Lower-Middle-Income OR Lower Middle Income OR Low Middle Income OR Low-Middle-Income<br>AND<br>Refer* OR Assign* OR Introduc* OR Prescribe* OR Uptak* OR Recruit* OR Participat* OR Attend* OR Complet* OR Finish* OR Adhere*
CINAHL	((MH "Pulmonary Disease, Chronic Obstructive+") OR (MH "Lung Diseases, Obstructive+")) OR Chronic obstructive pulmonary disease* OR Chronic obstructive airway disease* OR Chronic obstructive lung disease* OR COPD OR COAD OR COLD OR Chronic airflow obstruction* OR Obstructive lung disease* OR Obstructive pulmonary disease* OR Obstructive airway disease*<br>AND<br>(MH "Respiratory Therapy+") OR (MH "Breathing Exercises+") OR (MH "Respiratory Therapy+") OR (MH "Physical Therapy+") OR (MH "Rehabilitation, Pulmonary+") OR Respiratory Therap* OR Pulmonary Rehabilitat* OR Cardiopulmonary Rehabilitat* OR Pulmonary exercis* OR Breathing exercis* OR Physical therap*<br>AND<br>(MH "Low and Middle Income Countries") OR (MH "Developing Countries") OR (MH "Poverty+") OR Developing Countr* OR Developing Nation* OR LMIC* OR MIC* OR LIC* OR Low and Middle Income OR Low-and-Middle Income OR Low Income OR Low-income OR Middle Income OR Middle-income OR Lower-Middle-Income OR Lower Middle Income OR Low Middle Income OR Low-Middle-Income<br>AND<br>Refer* OR Assign* OR Introduc* OR Prescrib* OR Uptak* OR Recruit* OR Participat* OR Attend* OR Complet* OR Finish* OR Adhere*
Scopus	“Chronic obstructive pulmonary disease*” OR “Chronic obstructive airway disease*” OR “Chronic obstructive lung disease*” OR “COPD” OR “COAD”

	OR “COLD” OR “Chronic airflow obstruction*” OR “Obstructive lung disease*” OR “Obstructive pulmonary disease*” OR “Obstructive airway disease*”<br>AND<br>“Respiratory Therap*” OR “Pulmonary Rehabilitat*” OR “Cardiopulmonary Rehabilitat*” OR “Pulmonary exercis*” OR “Breathing exercis*” OR “Physical therap*”<br>AND<br>“Developing Countr*” OR “Developing Nation*” OR “LMIC*” OR “MIC*” OR “LIC*” OR “Low and Middle Income” OR “Low-and-Middle Income” OR “Low Income” OR “Low-income” OR “Middle Income” OR “Middle-income” OR “Lower-Middle-Income” OR “Lower Middle Income” OR “Low Middle Income” OR “Low-Middle-Income”<br>AND<br>Refer* OR Assign* OR Introduc* OR Prescribe* OR Uptak* OR Recruit* OR Participat* OR Attend* OR Complet* OR Finish* OR Adhere*

Table S3: Quality assessment of the included studies

	**Gushken *et al.* (1)**	**Xie *et al.* (2)**	**Yao et al. (3)**	**Sami *et al.* (4)**	**Betancourt-Peña *et al.* (5)**
Clearly stated research question	Unclear	Yes	Yes	Unclear	Yes
Clearly stated sample selection criteria	Unclear	Yes	Yes	Yes	Yes
Clear recruitment method	Unclear	Yes	Yes	Unclear	Yes
Adequately described sample characteristics	No	Yes	Yes	Yes	Yes
Appropriate and adequate sample size	Unclear	Yes	Yes	Unclear	Yes
Adequately described data collection method	Yes	Yes	Yes	Yes	Yes
Systematic data collection	Yes	Yes	Yes	Yes	Yes
Clear researcher–patient relationship	No	No	Yes	No	Yes

Appropriate data analysis method	Yes	Yes	Yes	Yes	Yes
Evidence to support analysis	Yes	Yes	Yes	Yes	Yes
Efforts to establish validity	No	No	Yes	Yes	No
Appropriate conclusion	Yes	Unclear	Yes	Yes	Yes
Total	7/12	9.5/12	12/12	9.5/12	11/12

Table S4: Studies that reported barriers and enablers to referral, uptake, and completion of PR

**Study**	**Referral to PR**		**Uptake of PR**		**Attendance and completion of PR**	
	Barrier	Enabler	Barrier	Enabler	Barrier	Enabler
**Gushken *et al.* (1)**	Yes	Yes	No	No	No	No
**Xie *et al.* (2)**	No	No	Yes	Yes	No	No
**Yao *et al.* (3)**	No	No	Yes	Yes	No	No
**Sami *et al.* (4)**	No	No	Yes	No	Yes	Yes
**Betancourt-Peña *et al.* (5)**	No	No	No	No	Yes	Yes

References:

1. Gushken F, Degani-Costa LH, Colognese TCP, Rodrigues MT, Zanetti M, Bonamigo-Filho JL, et al. Barriers to enrollment in pulmonary rehabilitation: medical knowledge analysis. Einstein (Sao Paulo). 2021;19:eAO6115. Epub 20211025. doi: 10.31744/einstein_journal/2021AO6115. PubMed PMID: 34705946; PubMed Central PMCID: PMC8522705.
2. Xie L, Liu Z, Hao S, Wu Q, Sun L, Luo H, et al. Assessment of knowledge, attitude, and practice towards pulmonary rehabilitation among COPD patients: A multicenter and cross-sectional survey in China. Respir Med. 2020;174:106198. Epub 20201020. doi: 10.1016/j.rmed.2020.106198. PubMed PMID: 33120194.
3. Yao XM, Li JM, He JL, Zhang QZ, Yu Y, He YA, et al. A Kano model-based demand analysis and perceived barriers of pulmonary rehabilitation interventions for patients with chronic obstructive pulmonary disease in China. Plos One. 2023;18(12). doi: 10.1371/journal.pone.0290828. PubMed PMID: WOS:001130043100009.

4. Sami R, Salehi K, Hashemi M, Atashi V. Exploring the barriers to pulmonary rehabilitation for patients with chronic obstructive pulmonary disease: a qualitative study. BMC Health Serv Res. 2021;21(1):828. Epub 20210817. doi: 10.1186/s12913-021-06814-5. PubMed PMID: 34404393; PubMed Central PMCID: PMC8369747.
5. Betancourt-Peña J, Ávila-Valencia JC, Rodríguez-Castro J. Adherence to Pulmonary Rehabilitation in Patients with Chronic Obstructive Pulmonary Disease (COPD). Journal of Respiration. 2023;3(3):130-40. doi: 10.3390/jor3030013.
